# Reliability of the Radiographic Sagittal and Frontal Tibiotalar Alignment after Ankle Arthrodesis

**DOI:** 10.1371/journal.pone.0154224

**Published:** 2016-04-28

**Authors:** Madeleine Willegger, Johannes Holinka, Elena Nemecek, Peter Bock, Axel Hugo Wanivenhaus, Reinhard Windhager, Reinhard Schuh

**Affiliations:** 1 Department of Orthopaedics, Vienna General Hospital, Medical University of Vienna, Vienna, Austria; 2 Foot and Ankle Unit, Orthopaedic Hospital Vienna-Speising, Vienna, Austria; Kanazawa University, JAPAN

## Abstract

**Background:**

Accurate measurement of the tibiotalar alignment is important in radiographic outcome assessment of ankle arthrodesis (AA). In studies, various radiological methods have been used to measure the tibiotalar alignment leading to facultative misinterpretation of results. However, to our knowledge, no previous study has investigated the reliability of tibiotalar alignment measurement in AA. We aimed to investigate the reliability of four different methods of measurement of the frontal and sagittal tibiotalar alignment after AA, and to further clarify the most reliable method for determining the longitudinal axis of the tibia.

**Methods:**

Thirty-eight weight bearing anterior to posterior and lateral ankle radiographs of thirty-seven patients who had undergone AA with a two screw fixation technique were selected. Three observers measured the frontal tibiotalar angle (FTTA) and the sagittal tibiotalar angle (STTA) using four different methods. The methods differed by the definition of the longitudinal tibial axis. Method A was defined by a line drawn along the lateral tibial border in anterior to posterior radiographs and along the posterior tibial border in lateral radiographs. Method B was defined by a line connecting two points in the middle of the proximal and the distal tibial shaft. Method C was drawn „freestyle”along the longitudinal axis of the tibia, and method D was defined by a line connecting the center of the tibial articular surface and a point in the middle of the proximal tibial shaft. Intra- and interobserver correlation coefficients (ICC) and repeated measurement ANOVA were calculated to assess measurement reliability and accuracy.

**Results:**

All four methods showed excellent inter- and intraobserver reliability for the FTTA and the STTA. When the longitudinal tibial axis is defined by connecting two points in the middle of the proximal and the distal tibial shaft, the highest interobserver reliability for the FTTA (ICC: 0.980; CI 95%: 0.966–0.989) and for the STTA (ICC: 0.997; CI 95%: 0.996–0.999) is provided. Intergroup analysis for FTTA measurements revealed a statistically significant difference between the method in which the lateral border of the tibia was used to determine the longitudinal axis of the tibia, and the other methods in which the longitudinal axis was defined by bisecting the tibia.

**Conclusions:**

When the longitudinal axis of the tibia is defined by connecting two points in the middle of the proximal and the distal tibial shaft for measuring the FTTA and STTA, the most favorable interobserver reliability is provided. Therefore, this method can be recommended for evaluating the frontal and the sagittal alignment on anterior to posterior and lateral radiographs after ankle arthrodesis.

## Introduction

Ankle arthrodesis (AA) is currently the most commonly performed surgical reconstruction procedure for the treatment of debilitating end-stage ankle arthritis. The appropriate position of AA in the sagittal and frontal plane is essential for its clinical long-term success.[1–5] Malalignment following tibiotalar fusion is recognized to be an underlying cause of persisting chronic pain in the hindfoot. [1,4,6,7] Plantarflexion malalignment decreases the sagittal motion of the foot[1,2,8] and varus tibiotalar fusion shifts the loading axis on the lateral side of the foot.[1] These adverse biomechanical conditions lead to abnormal gait patterns and to increased hindfoot symptoms.[1,4,6,9–11]

Alignment measurement in fused ankles directs decision making in symptomatic patients considering conservative treatment or revision surgery. Therefore an accurate radiographic measurement of the position of the tibiotalar alignment in two planes, i.e. the frontal and the sagittal tibiotalar angle (FTTA and STTA) has become an important outcome assessment. [1–4,10,12–16]

Various radiological methods have been used to record sagittal and frontal alignment after ankle arthrodesis (AA) and total ankle replacement (TAR).[2,12,14,15,17–20] To date, no precise methodology exists for measuring the FTTA and STTA. Due to different non standard methods of measurement, reliable comparisons between follow-up studies are difficult to be performed. This leads to inconsistent results in angular measurements and to a potential under- or overestimation of a malalignment. Despite the importance of tibiotalar alignment, its radiographic measurement accuracy and reproducibility is unclear. However, to the best of our knowledge, no previous study has investigated the reliability of the tibiotalar alignment measurement in AA.

The purpose of the present study was to investigate the inter- and intraobserver reliability of four different methods used to measure the frontal and the sagittal tibiotalar alignment after ankle arthrodesis and to further clarify the most reliable method for determining the longitudinal axis of the tibia. We hypothetizised that the frontal and the sagittal tibiotalar angle would differ between the measurement methods.

## Materials and Methods

**Fig 1:**
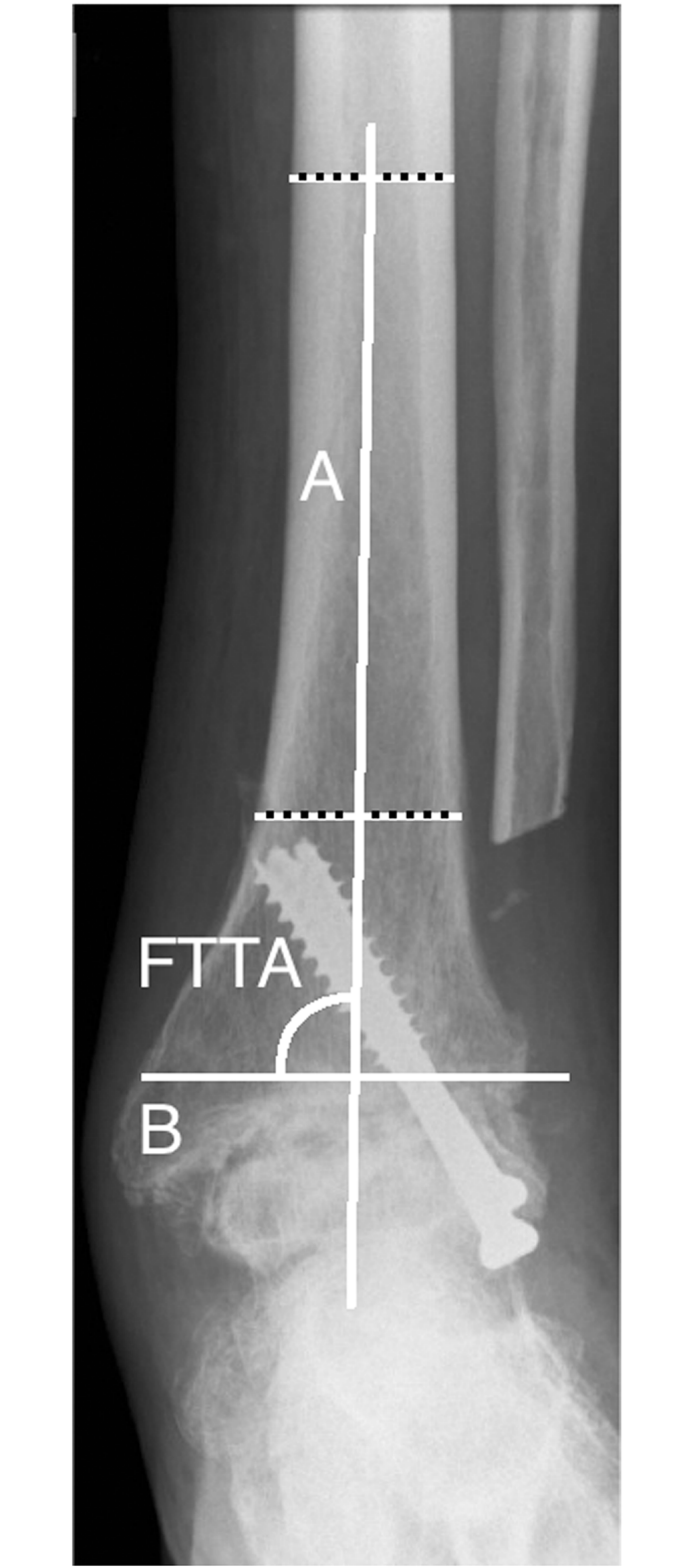
Anterior-posterior radiograph ankle arthrodesis. Anterior to posterior ankle radiograph with the measurement of the frontal tibiotalar angle (FTTA). The FTTA is the superomedial angle between the two axes A and B. A = longitudinal axis of the tibia, created by connecting two points in the middle of the proximal and the distal tibial shaft (according to method B). B = axis of the talus, defined by a line drawn through the talar shoulders.

**Fig 2:**
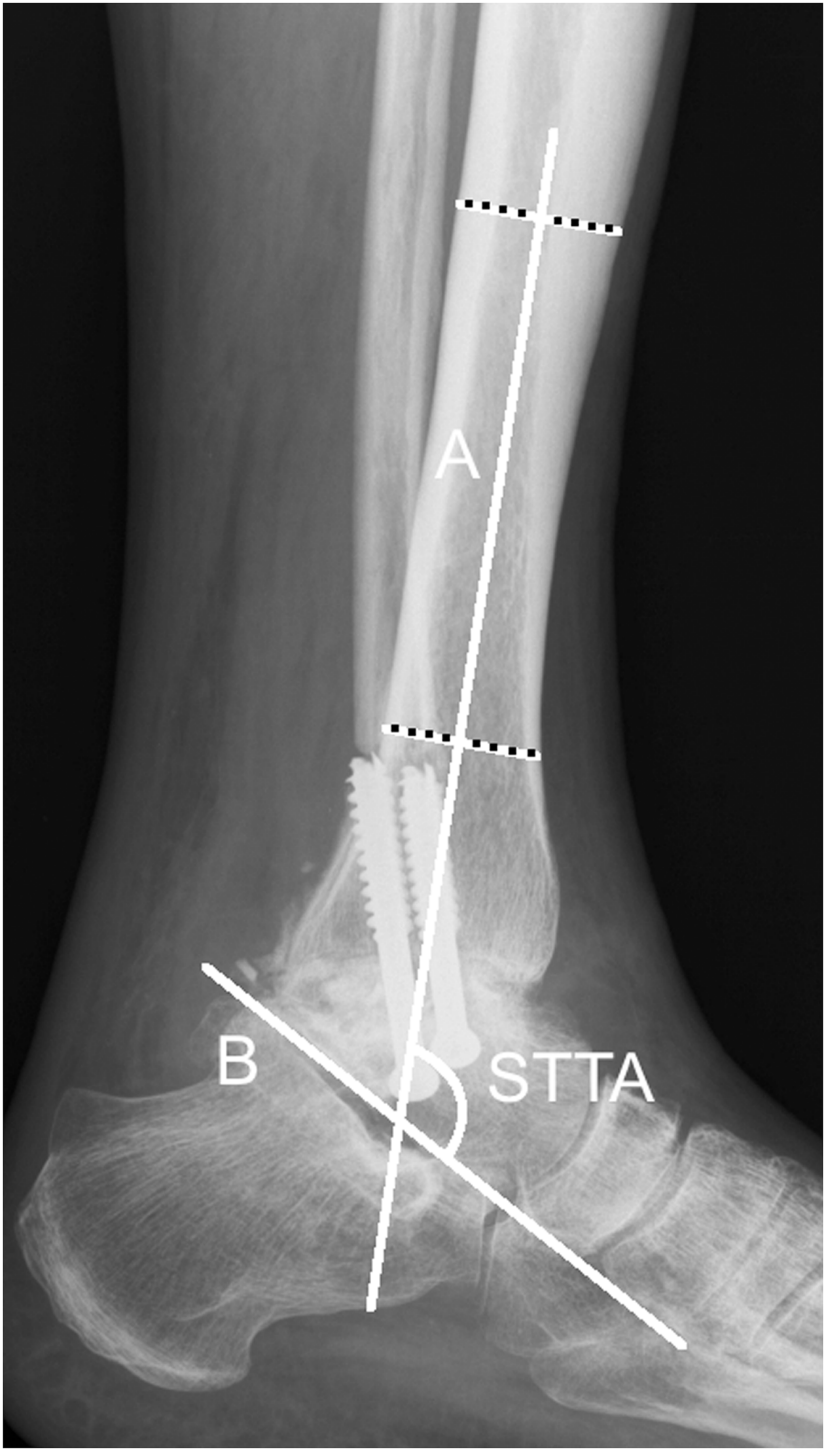
Lateral radiograph ankle arthrodesis. Lateral ankle radiograph with the measurement of the sagittal tibiotalar angle (STTA). The STTA is the angle between the the two axes A and B. A = longitudinal axis of the tibia, created by connecting two points in the middle of the proximal and the distal tibial shaft (according to method B). B = axis of the talus, defined by a line drawn from the inferior aspect of the posterior tubercule of the talus to the most inferior aspect of the talar neck.

This study was approved by the institutional review board (IRB) of the Medical University of Vienna (EK– 2002/2013). We retrospectively identified patients from a review of the medical records who underwent isolated ankle arthrodesis for end-stage ankle arthritis. Patient data had been anonymized prior to further analysis. Anterior to posterior and lateral standing weight bearing ankle radiographs were routinely obtained for radiographic follow-up at the same institution following a standardized protocol. All tibiotalar fusions were performed between 2006 and 2013 using a lateral surgical approach with a two screw fixation technique.[16] (Figs 1 and 2) Radiographs of patients who underwent a revision ankle arthrodesis or an additional fusion of the subtalar or talonavicular joint had been excluded.

Standard anterior to posterior and lateral radiographs of 38 fused ankles (20 left, 18 right ankles) in 17 female and 20 male patients were finally retrieved and investigated. The underlying etiology of ankle arthritis had been post traumatic in 23 ankles (60,5%), secondary in 12 ankles (31,6%), and primary in 3 ankles (7,9%), respectively. Secondary ankle arthritis included patients suffering from rheumatoid arthritis (n = 5), avascular talus necrosis (n = 3), hemophilia (n = 2), osteochondritis dissecans (n = 1), and clubfoot (n = 1). Patient age at operation ranged from 23 to 73 years (mean age: 53,7 years). Radiographs were recorded after a minimum of six months post operatively to assert a bony union of the arthrodesis.

Anterior to posterior radiographs have been acquired with the foot in 15 degrees internal rotation. Lateral radiographs have been taken with the ankle parallel to the film with a field of view that included the midshaft of the tibia to below the calcaneus. The beam was focused on the center of the ankle joint with a source-to-image distance of 125 cm at 51 kV and 12,5 mAs (Multix Fusion, Siemens, Erlangen, Germany).

One of the authors (R.S.) drew the axis of the talus, after which three investigators (J.H., E.N., M.W.), an attending foot and ankle surgeon with eight years of experience, a senior orthopaedic resident (6th year of residency), and a final year medical student (6th year of medical school), independently determined the longitudinal axis of the tibia on a/p and lateral radiographs. Furthermore, to ensure that the measurements were precise and reproducible, the longitudinal axis of the talus was not erased from the images until all of the radiographic assessments had been finished.

### Radiographic Measurements

**Fig 3:**
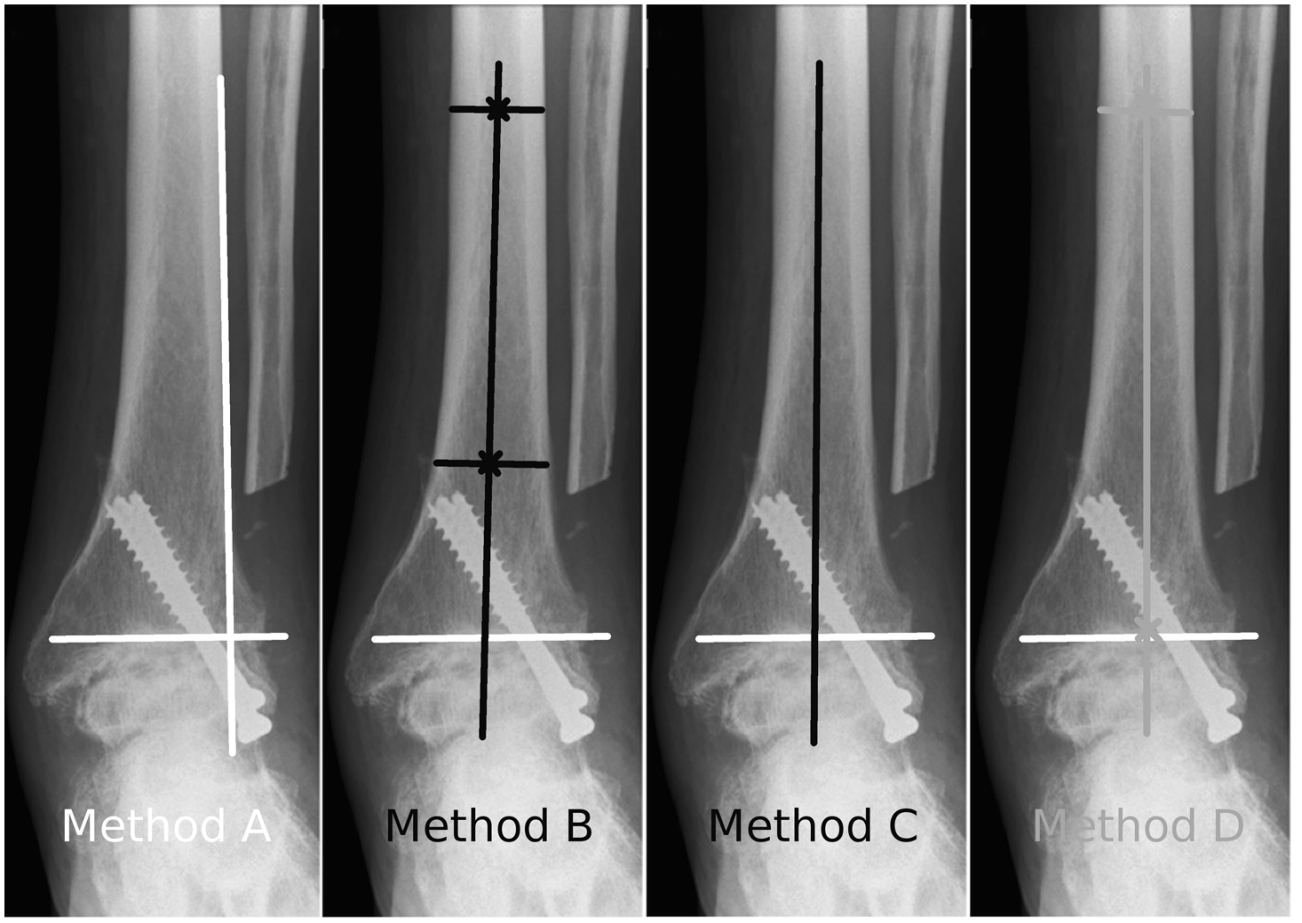
Measurement methods for the FTTA. Diagrams depicting the four different methods for drawing the longitudinal axis of the tibia for FTTA measurement on anterior to posterior radiographs.

Three preexisting methods and a new method of determination of the longitudinal tibial axis on a/p radiographs have been used (Fig 3):

Method A: A line is drawn along the lateral border of the tibia. [6,17]
Method B: A line is drawn by connecting two points in the middle of the proximal and the distal tibial shaft.[19]
Method C: A line is drawn along the the longitudinal axis of the tibia. The investigator is asked to draw it „freestyle“.[18,21]
Method D: A line is drawn to connect the center of the tibial articular surface and a point in the middle of the proximal tibial shaft.

The axis of the talus was defined as a line drawn through the talar shoulders on anterior to posterior radiographs. [14,15] The superomedial angle between these two axes defined the FTTA. (Fig 1)

**Fig 4:**
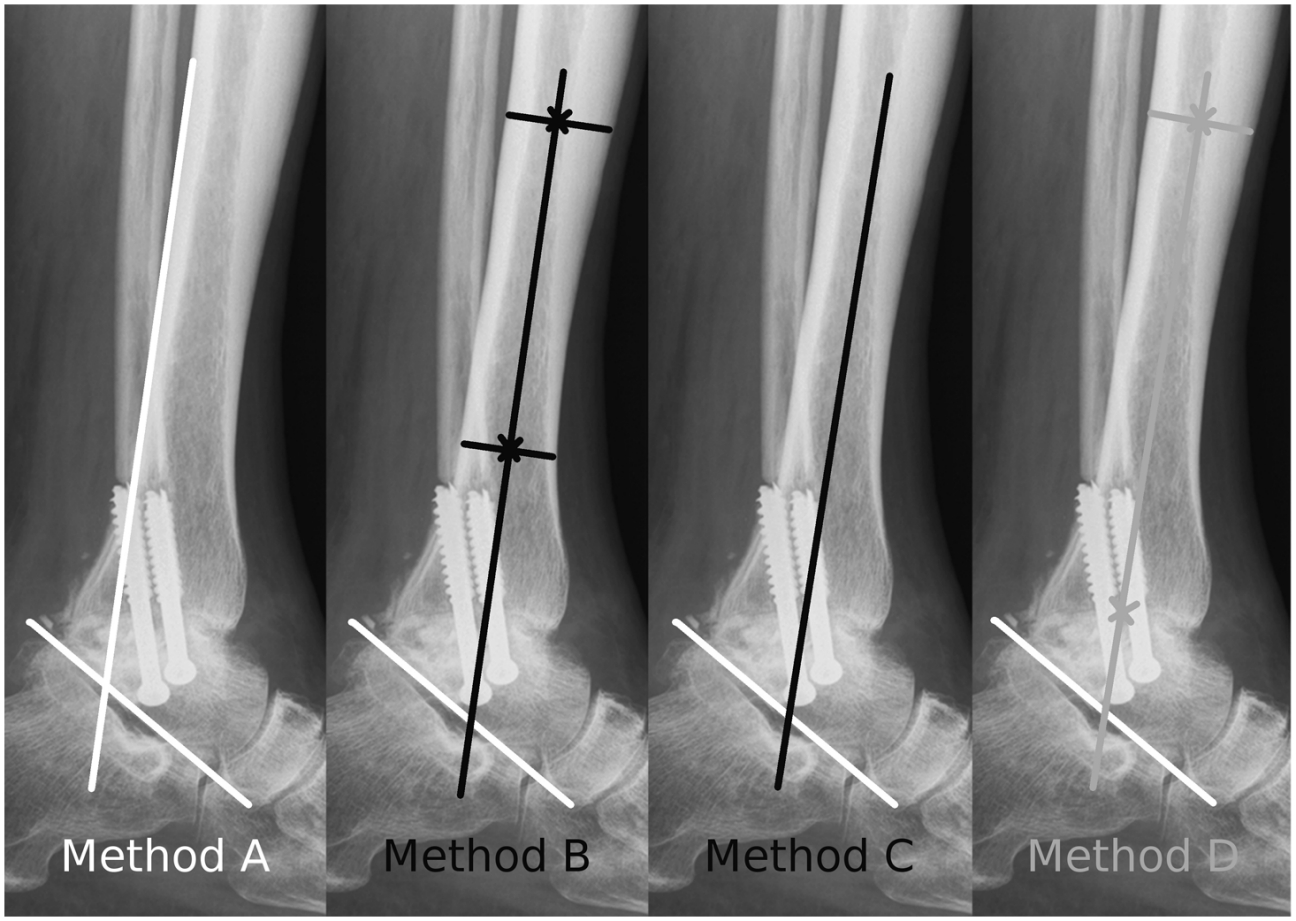
Measurement methods for the STTA. Diagrams depicting the four different methods for drawing the longitudinal axis of the tibia for STTA measurement on lateral ankle radiographs.

For the evaluation of the sagittal tibiotalar angle (STTA) on lateral ankle radiographs, we likewise used three described methods and a newly introduced method for determining the longitudinal axis of the tibia (Fig 4):

Method A: A line is drawn along the posterior border of the tibia.[17]
Method B: A line is drawn by connecting two points in the middle of the proximal and distal tibial shaft.[19]
Method C: A line is drawn along the longitudinal axis of the tibia. The investigator is asked to draw it „freestyle“.[18,21]
Method D: A line is drawn to connect the center of the tibial articular surface and a point in the middle of the proximal tibial shaft.

On lateral radiographs the axis of the talus was determined by drawing a line from the inferior aspect of the posterior tubercule of the talus to the most inferior aspect of the talar neck.[2] The angle formed by these two axes defined the STTA. (Fig 2)

All radiographic measurements were performed with the use of a picture archiving and communication system (PACS) (IMPAX; Agfa HealthCare, Mortsel, Belgium) software.

### Assessment of reliability and accuracy

Reliability was defined as the consistency of the measurements, and accuracy was defined as the proximity of angular measurements between the methods. The radiographic assessment was performed by three observers in two sessions, with each of the four different methods at a minimum interval of one week (mean 1.2 weeks; range 1 to 2 weeks). [22] Intraobserver reliability was assessed on the basis of the ICC of each method for each observer. Interobserver reliability was assessed on the basis of the agreement among the three observers for each method. The first measurement obtained by each observer was used for the analysis of interobserver reliability. Each observer was blinded to results assigned by the other observers and to patient data. Radiographs were presented to each observer in random order by a research assistant who did not participate in the reliability and accuracy sessions.

In order to assess the accuracy of FTTA and STTA measurements the mean differences of the measurements between the methods have been compared.

### Statistical analysis

Prior to the medical records review a sample size analysis was conducted in order to determine the minimum number of ankles required to obtain sufficient statistical power. In this study, the minimum sample size for reliability was calculated as 36 ankles by setting the intraclass correlation coefficient (ICC) target as 0.8, 95% confidence interval as 0.2, and number of observers as three with a Bonett’s approximation.[23]

The intraclass correlation coefficient (ICC) and their 95% confidence intervals were calculated for all continuous variables within and between observers, and the data were used to estimate the intraobserver and interobserver reliability. The ICCs were calculated in the setting of a two-way random-effect model, assuming a single measurement and absolute agreement. Intraobserver and interobserver reliability were classified as minimal (correlation coefficient, < 0.25), low (0.26 to 0.49), moderate (0.50 to 0.69), high (0.70 to 0.89), or excellent (> 0.90).[24]

The accuracy of the FTTA and STTA measurement methods were analyzed by calculating intergroup differences using repeated measurement ANOVA. For ANOVAs that demonstrated a statistically significant difference, a post hoc Tukey Honestly Significant Difference test was conducted to assess the location of the means that were statistically significant between the methods.

All analyses were performed using SPSS 20.0 for Windows XP (SPSS Inc, Chicago, IL, USA), and the level of significance was set at P < .05.

## Results

### Reliability

The mean values for the frontal tibiotalar angle (FTTA) and the sagittal tibiotalar angle (STTA) are illustrated in Table 1.

**Table 1:** Radiographic Measurements.

		mean	range	SD	95%CI (lower—upper)
a/p (FTTA)	A	88,1588	78,2–98,7	3,43193	87,71–88,60
	B	89,5890	81,3–97,9	2,97035	89,20–89,97
	C	89,0399	79,9–96,8	3,00734	88,64–89,43
	D	89,4632	80,8–95,6	2,76115	89,10–89,82
	all	89,0627	78,2–98,7	3,09850	88,86–89,26
lateral (STTA)	A	111,9610	94,0–129,7	9,12038	110,77–113,15
	B	112,6864	93,8–130,9	8,88165	111,52–113,84
	C	112,3031	93,2–130,0	8,89369	111,14–113,46
	D	112,3715	94,7–130,5	8,42659	111,27–113,47
	all	112,3305	93,2–130,9	8,82338	111,75–112,90

This table shows the mean values in degrees with standard deviation (SD) and 95% confidence intervals (95% CI) of FTTA and STTA measurements for all four methods.

All four methods showed excellent reliability for the measurement of the FTTA and the STTA for interobserver reliability. The interobserver coefficient (ICC) of repeatability for the FTTA was 0.968 (CI 95% 0.945–0.982) for method A, 0.980 (CI 95% 0.966–0.989) for method B, 0.954 (CI 95% 0.921–0.974) for method C, and 0.949 (CI 95% 0.907–0.972) for method D. The interobserver coefficient (ICC) of repeatability for the STTA was 0.996 (CI 95% 0.992–0.998) for method A, 0.997 (CI 95% 0.996–0.999) for method B, 0.996 (CI 95% 0.994–0.998) for method C, and 0.996 (CI 95% 0.993–0.998) for method D. Method B yielded the highest interobserver reliability for the FTTA and the STTA. (Table 2)

**Table 2:** Inter- and Intraobserver Reliability.

ICC interobserver reliability								
	method 1	95% CI	method 2	95% CI	method 3	95% CI	method 4	95% CI
ap (FTTA)	0,968	0,945–0,982	***0*,*98***	0,966–0,989	0,954	0,921–0,974	0,949	0,907–0,972
lat (STTA)	0,996	0,992–0,998	***0*,*997***	0,996–0,999	0,996	0,994–0,998	0,996	0,993–0,998
ICC intraobserver reliability								
	method 1	95% CI	method 2	95% CI	method 3	95% CI	method 4	95% CI
observer 1 ap	***0*,*994***	0,988–0,997	0,982	0,965–0,990	0,985	0,971–0,992	0,958	0,919–0,978
observer 1 lat	***0*,*998***	0,996–0,999	0,997	0,995–0,999	0,995	0,990–0,997	0,997	0,993–0,998
observer 2 ap	0,927	0,859–0,962	0,963	0,930–0,981	0,951	0,905–0,975	***0*,*965***	0,905–0,975
observer 2 lat	0,994	0,989–0,997	***0*,*996***	0,992–0,998	0,995	0,990–0,997	0,993	0,987–0,997
observer 3 ap	***0*,*981***	0,964–0,990	0,978	0,958–0,988	0,912	0,830–0,954	0,97	0,943–0,984
observer 3 lat	***0*,*998***	0,996–0,999	0,997	0,994–0,998	0,996	0,993–0,998	0,992	0,984–0,996

Inter- and intraobserver reliability of the FTTA and STTA as determined with use of Intraclass Correlation Coefficients (ICC) with 95% confidence intervals (95% CI). The values are given separately according to the four methods for drawing the longitudinal axis of the tibia as described in the text. The highest ICCs for inter- and interobserver reliability are highlighted in bold/italic.

Intraobserver reliability for the FTTA revealed excellent reliability for all methods in observer 1 (ICC range 0.958–0.994), observer 2 (ICC range 0.927–0.965), and observer 3 (ICC range 0.912–0.981). Reliability for the STTA showed excellent intraclass correlation (ICC) for all methods in observer 1 (range 0.995–0.998), observer 2 (range 0.993–0.996), and observer 3 (range 0.992–0.998). For observers 1 and 3, method A yielded the highest intraobserver correlation coefficients in FTTA and STTA measurement. Observer 2 reached the highest ICC for FTTA with method D and for STTA with method B, respectively. (Table 2)

**Fig 5:**
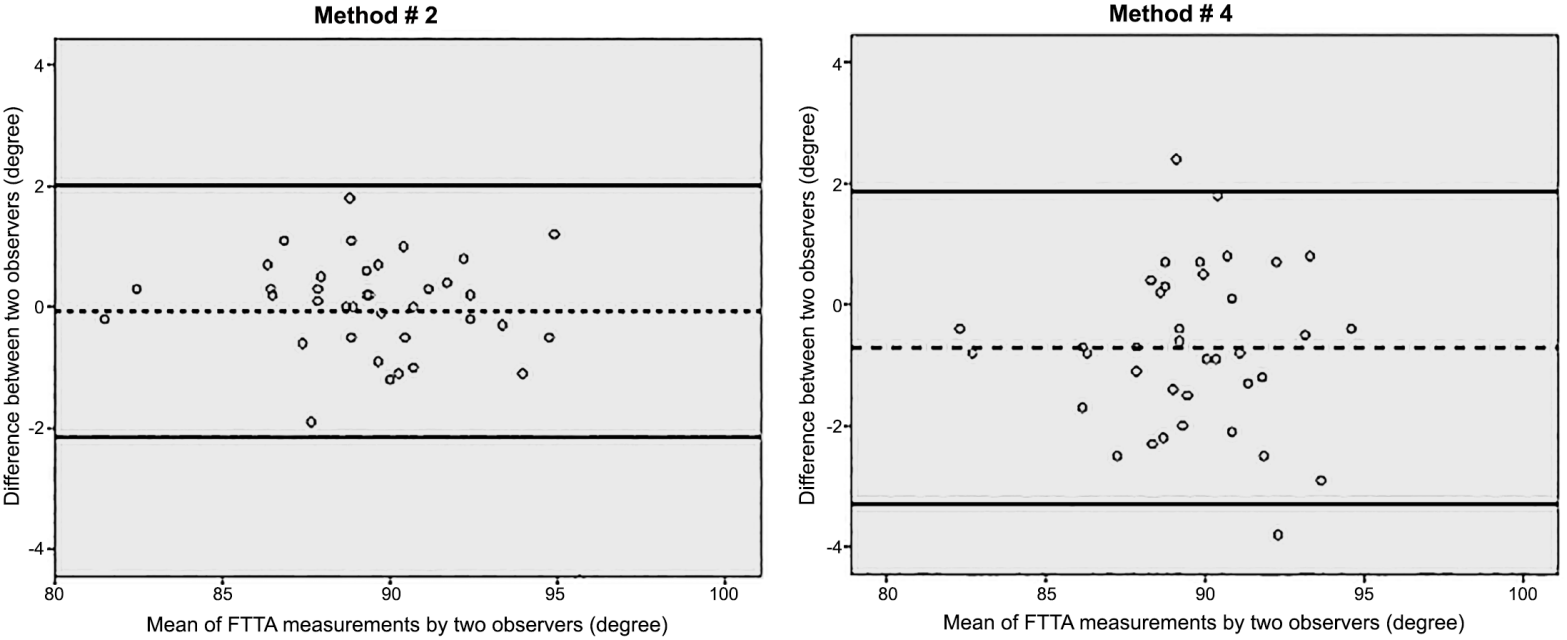
Bland Altman Plots. Bland and Altman plots depicting the FTTA measurements of method 2 and 4, demonstrating better agreement with use of method 2 (left) compared with use of method 4 (right). The average of the measurements made by two observers is plotted against the difference between the measurements made by those two observers. The dashed lines represent the mean value of all differences between the two observers, and the dotted lines represent the 95% limits of agreement.

A graphic representation of differences in the interobserver agreement for FTTA measurement under usage of method 2 and 4 is shown in Fig 5. [25]

### Accuracy

The mean difference in FTTA between the methods was -1.43 degrees (95%CI: -2.16 to -0.69) for method A and B, -0.88 degrees (95%CI: -1.61 to -0.14) for method A and C, and -1.30 degrees (95%CI: -2.04 to -0.56) for method A and D, respectively. Intergroup analysis for FTTA measurements on a/p radiographs revealed statistically significant mean differences in method A compared to method B (P < .000), C (P < .011), and D (P < .000). In terms of the STTA measurements the mean differences were not significantly different between the methods. (Table 3)

**Table 3:** Measurement Accuracy.

a/p FTTA				
		mean difference	p-value	95%CI (lower—upper)
Method A	B	-1,43026*	,000	-2,16 to -0,69
	C	-,88114*	,011	-1,61 to -0,14
	D	-1,30439*	,000	-2,04 to -0,56
Method B	A	1,43026*	,000	0,69 to -2,16
	C	,54912	,220	-0,18 to -1,28
	D	,12588	,971	-0,60 to -0,86
Method C	A	,88114*	,011	0,14 to -1,61
	B	-,54912	,220	-1,28 to -0,18
	D	-,42325	,450	-1,15 to 0,31
Method D	A	1,30439*	,000	0,56 to -2,04
	B	-,12588	,971	-0,86 to 0,60
	C	,42325	,450	-0,31 to 1,15
Lateral STTA				
		mean difference	p-value	95%CI (lower—upper)
Method A	B	-,72544	,817	-2,85 to 1,40
	C	-,34211	,976	-2,47 to 1,78
	D	-,41053	,960	-2,54 to 1,71
Method B	A	,72544	,817	-1,40 to 2,85
	C	,38333	,967	-1,74 to 2,51
	D	,31491	,981	-1,81 to 2,44
Method C	A	,34211	,976	-1,78 to 2,47
	B	-,38333	,967	-2,51 to 1,74
	D	-,06842	1,000	-2,19 to 2,06
Method D	A	,41053	,960	-1,71 to 2,54
	B	-,31491	,981	-2,44 to 1,81
	C	,06842	1,000	-2,06 to 2,19

The mean differences in FTTA and STTA measurements between the four methods are outlined in this table. Statistically significant results from intergroup analysis are marked with *. Exact p-values as well as upper and lower 95% confidence intervals are given.

## Discussion

Exact assessment of tibiotalar alignment in ankle arthrodesis is crucial for surgical decision making in patients with persisting hindfoot pain. However, there is no consensus on the radiographic alignment measurement methodology following AA.

In the present study, we investigated the inter- and intraobserver reliability of the frontal (FTTA) and the sagittal tibiotalar angle (STTA) as well as the measurement accuracy following AA, thus permitting us to assess the most reliable method and consider its application for future AA follow-up. We further compared four different methods to measure the FTTA and STTA. These methods differ by the determination of the longitudinal axes of the tibia. All investigated methods revealed excellent interobserver reliability. However, method B, in which the longitudinal axis of the tibia was defined by a line connected by two points bisecting the tibial shaft distal and proximal[19], was the most reliable method for FTTA (0.980; CI 95% 0.966–0.989) assessment. In lateral ankle radiographs the highest interobserver reliability for STTA measurements is again provided by using method B (0.997, CI 95% 0.996–0.999). Intraobserver reliability showed excellent intraclass correlations for FTTA and STTA measurements for all three observers. Method A revealed the most reliable results for intraobserver reliability in observer 1 and 3 for FTTA and STTA measurement. Observer 2 reached the highest intraobserver reliability for FTTA with method D and for STTA with method B.

Intergroup analysis comparing the resulting angles of the FTTA measurement between the methods revealed statistically significant differences of the mean. Indeed, method A, in which the longitudinal axis of the tibia was drawn by a line along the lateral border of the tibia[17] differed statistically from method B (P < .000), C (P < .011), and D (P < .000). This finding implies that even though all methods of FTTA measurement showed excellent reliability, there is a significant difference in definite angular outcome measurement according to the frontal tibiotalar alignment. No statistically significant difference was present for measurement methods of STTA.

We conclude that in a/p radiographs the longitudinal axis of the tibia is not adequately represented by the lateral border of the tibia. However, observers 1 and 3 produced the most reliable results with method A for FTTA and STTA measurement. Obviously the lateral and posterior borders of the tibia represent a good reference point for angular measurements, but in accordance with interobserver reliability and comparison of the mean differences, method B showed the most reliable results. Therefore we recommend to use method B for future radiographic AA alignment measurements.

Clinical management and surgical treatment of hindfoot pathology requires accurate and reliable radiographic assessment of deformities. Barg et al. evaluated the diagnostic agreement of the medial distal tibial angle (MDTA) measurement in the mortise view compared to the hindfoot alignment view. They showed a substantial disagreement in primary alignment assessment between the mortise and hindfoot alignment view as quantified by the MDTA.[26] In foot and ankle surgery reliability of certain angular measurements has already been established for several deformities[19,22,27–33], but following AA or TAR validated radiographic methods have only scarcely been evaluated for measurement reliability. [17,34–39] Pyevich et al. defined a five degree criterion for determining migration of the tibial or talar component after reliability testing of the angular measurements to assess component alignment in TAR. To the best of our knowledge, we evaluated reliability in radiographic alignment measurement following AA for the first time.

There are several limitations associated with the present study. First, the methods for FTTA and STTA measurement only differed by the determination of the longitudinal axes of the tibia. The talar axis was drawn by the senior author according to a previously published method. [2,14,15] The most reliable method for measuring the talar axis has not yet been defined. Secondly, the talar shoulders for determining the talar axis in a/p radiographs are not easy to identify due to prior fusion of the joint. Adjacent subtalar joint arthritis may affect the determination of the sagittal talar axis. Third, the rotational element in AA was not assessed in the a/p and lateral radiographic methods but it is as well a contributing factor in AA positioning.[2] Additionally the position and rotation of the ankle during standing radiograph as well as the tilt of the radiographic beam can affect projection on ankle radiographs which further leads to altered angular ankle or hindfoot measurements. [38,40] To avoid a potential incorrect assessment all radiographs have been performed at a single institution following a standardized protocol. Fourth, although sample size calculation has been performed and showed adequate amount of the evaluated ankle radiographs to obtain sufficient statistical power, a relatively small number of ankles had been included in this study. These limitations must be taken into consideration when our results are applied in clinical practice.

In conclusion, the present study revealed excellent inter- and intraobserver reliability for the radiographic assessment of the tibiotalar alignment in ankle arthrodesis. Since general accepted recommendations for the evaluation of FTTA and STTA in AA do not exist, we established reliability analysis for the first time. We subsequently compared the mean differences of four measurement methods and defined the most reliable method for drawing the longitudinal axis of the tibia. According to the results of the present study, we recommend method B for future radiographic AA alignment measurement. With this method the longitudinal axis of the tibia is drawn by connecting two points in the middle of the proximal and the distal tibial shaft for measuring the FTTA and STTA. The use of a standard alignment measurement methodology allows for plausible comparison and evaluation of radiographic outcome in different patient cohorts. Based on the findings of our study, criteria for post-operative radiographic malalignment in ankle arthrodesis could be established.
